# Anatomical and Technical Factors Influence the Rate of In-Stent Restenosis following Carotid Artery Stenting for the Treatment of Post-Carotid Endarterectomy Stenosis

**DOI:** 10.1371/journal.pone.0161716

**Published:** 2016-09-09

**Authors:** Marine Gaudry, Jean-Michel Bartoli, Laurence Bal, Roch Giorgi, Mariangela De Masi, Pierre-Edouard Magnan, Philippe Piquet

**Affiliations:** 1 APHM, Hôpital Timone, Department of Vascular Surgery, 13005, Marseille, France; 2 APHM, Hôpital Timone, Department of Radiology, 13005, Marseille, France; 3 Aix-Marseille Univ, INSERM, IRD, SESSTIM, Sciences Economiques & Sociales de la Santé & Traitement de l’Information Médicale, Marseille, France; 4 APHM, Hôpital Timone, Service Biostatistique et Technologies de l’Information et de la Communication, Marseille, France; "INSERM", FRANCE

## Abstract

**Background:**

Carotid artery stenting (CAS) has been advocated as an alternative to redo surgery for the treatment of post-carotid endarterectomy (CEA) stenosis. This study analyzed the efficacy of CAS for post-CEA restenosis, focusing on an analysis of technical and anatomical predictive factors for in-stent restenosis.

**Methods:**

We performed a retrospective monocentric study. We included all patients who underwent CAS for post-CEA restenosis at our institution from July 1997 to November 2013. The primary endpoints were the technical success, the presence of in-stent restenosis >50% or occlusion, either symptomatic or asymptomatic, during the follow-up period, and risk factors for restenosis. The secondary endpoints were early and late morbidity and mortality (TIA, stroke, myocardial infarction, or death).

**Results:**

A total of 153 CAS procedures were performed for post-CEA restenosis, primarily because of asymptomatic lesions (137/153). The technical success rate was 98%. The 30-day perioperative stroke and death rate was 2.6% (two TIAs and two minor strokes), and rates of 2.2% (3/137) and 6.2% (1/16) were recorded for asymptomatic and symptomatic patients, respectively. The average follow-up time was 36 months (range, 6–171 months). In-stent restenosis or occlusion was observed in 16 patients (10.6%). Symptomatic restenosis was observed in only one patient. We found that young age (P = 0.002), stenosis > 85% (P = 0.018), and a lack of stent coverage of the common carotid artery (P = 0.006) were independent predictors of in-stent restenosis.

**Conclusion:**

We identified new risk factors for in-stent restenosis that were specific to this population, and we propose a technical approach that may reduce this risk.

## Introduction

The incidence of restenosis after carotid surgery varies from 1% to 36% [1–3], depending on the definition of restenosis and the length of follow-up. Surgery was the standard treatment for restenosis for many years, and the 30-day periprocedural stroke and death rate is lower than 3% in asymptomatic patients [4]. Redo carotid surgery is technically difficult and usually complicated by cranial nerve injury. In literature, the incidence of cranial nerve injuries has been reported to range from 1% to 17%, and studies show that most such reported injuries are transient. [5–9].

The development of endovascular techniques has considerably changed the indications for vascular surgery. Because the use of stents has extensively evolved and cerebral protection systems are frequently used, carotid angioplasty/stenting (CAS) has become an alternative method for treating carotid restenosis. Many studies have compared CAS to redo surgery [5–7, 10–12]. The short-term results of these studies have revealed that there is no significant difference between the two treatments [5–7, 9–13]. Endovascular treatment provides an advantage in that it reduces the risk of cranial nerve injury [5].

Restenosis after carotid endarterectomy (CEA) is an indication for CAS when the 30-day perioperative rate of stroke and death is less than 3% [14]. The expected benefit of this treatment is dependent on long-term anatomical results. Despite a low rate of periprocedural complications, in-stent restenosis after CAS is a frequent complication [2, 5, 11], especially when used to treat post-CEA restenosis [15, 16]. Few studies have analyzed the anatomical characteristics associated with lesions, which include residual stenosis, calcified lesions, the implantation of multiple stents, that predispose individuals to negative evolution [17–19].

The objective of this study was to determine the incidence of in-stent restenosis after CAS for the specific indication of post-CEA restenosis. Our study focused on an analysis of technical and anatomical predictive factors of in-stent restenosis.

## Methods

All of the patients included in this study were informed of the use of their data for clinical research.

All patient information was anonymized and de-identified prior to analysis.

The institutional review board approved this study. The president of the Ethical Committee of the French Society of Thoracic and Cardio-Vascular Surgery certified that the project had been assessed according to the current regulations framing clinical research in France.

This single-center retrospective study included 147 patients who underwent carotid angioplasty stenting at our institution from July 1997 to November 2013 to treat restenosis following CEA. Patients who were treated with carotid angioplasty for radiation-induced carotid stenosis or who had primary CAS were excluded from the study.

Clinical and demographic data and information on comorbidities and treatment were collected at admission.

Restenosis was defined as a diameter reduction >50% measured using computed tomography (CT) angiography or carotid angiography and a duplex scan.

In patients with post-CEA restenosis, the indications for CAS included symptomatic >50% and asymptomatic >80% restenosis. The symptomatic characteristics of post-CEA-restenosis included the occurrence of a transient ischemic attack (TIA) or stroke that was attributed to the lesion during the six months preceding the diagnosis of carotid restenosis.

All patients underwent initial imaging using CT angiography, which made it possible to evaluate the lesions (the North American Symptomatic Carotid Endarterectomy Trial [NASCET] criteria were used) [20] and cerebral vascularization and to anticipate the catheterization conditions (e.g., the type of aortic arch and the angulation of the supra-aortic trunks).

For this study, CT scans and/or angiographies were retrospectively analyzed by a radiologist at our institution. The lesion characteristics (degree of stenosis location density, the presence of calcification, dissection or ulceration, and length) and artery characteristics (ICA and CCA size and ICA and CCA elongation) were described.

**Fig 1:**
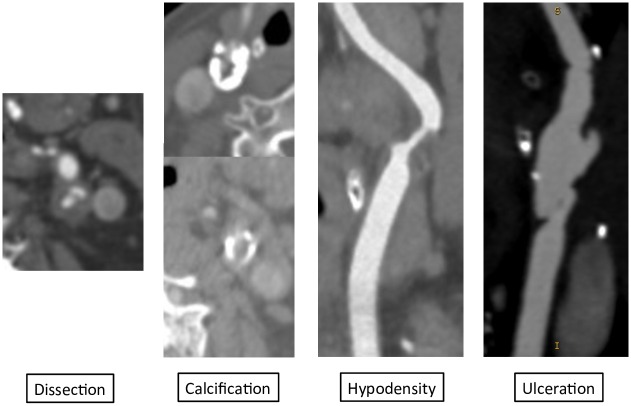
Lesion characteristics. Dissection, calcification, hypodensity and ulceration.

- Lesion description (Fig 1):
  - Hypodense: without any calcifications
  - Highly calcified: calcification > 50% of the total arterial circumference
  - Dissection: presence of an intimal flap
  - Ulceration: inhomogeneity or irregularity in the arterial wall

**Fig 2:**
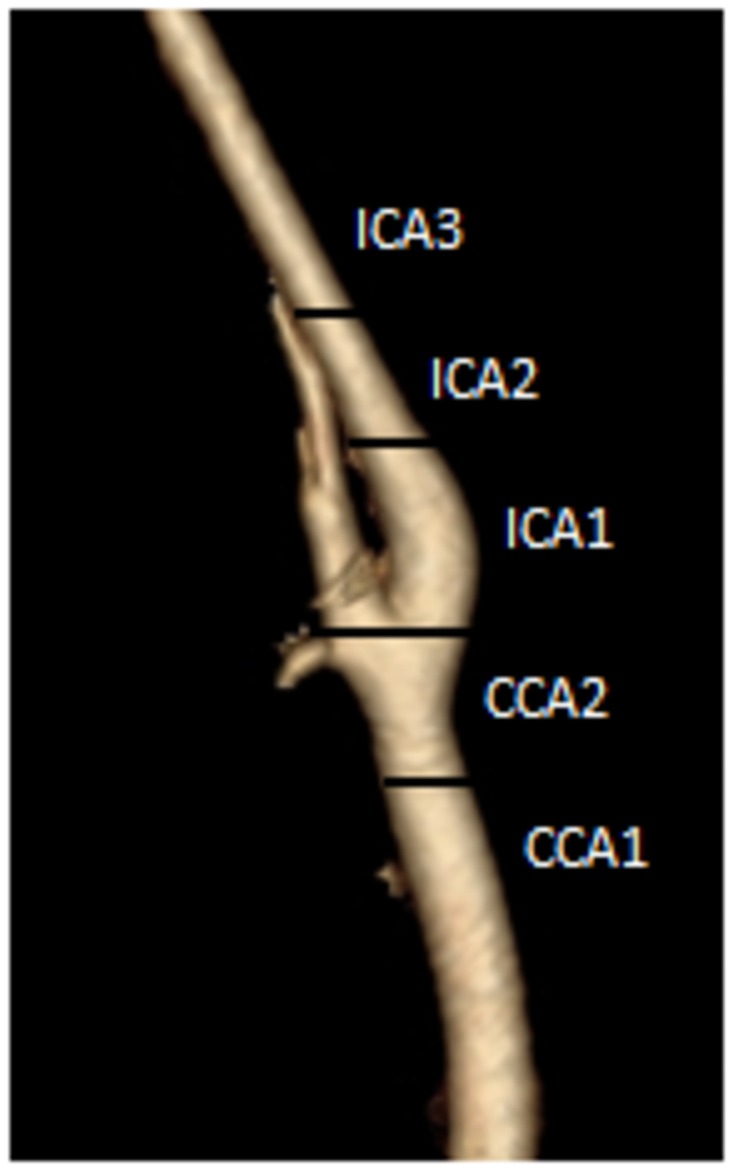
location of lesion. CCA = common carotid artery, ICA = internal carotid artery.

A classification system to describe the location and extent of the lesion was established (Fig 2). The number, diameter, and length of the stents that were used were reported.

### Procedure

All procedures were performed via a transfemoral approach under local anesthesia in our establishment by a team that consisted of both a vascular surgeon and an interventional radiologist. Selective angiography was performed to assess the degree of restenosis and intracranial circulation. The lesion was crossed with a 0.014” guide-wire. Since 2003, we have systemically used a cerebral protection system (0.014” FilterWire EZ^™^ system). In most cases, the stent is deployed without prior angioplasty. In-stent angioplasty (using a 4-6-mm-diameter balloon) was secondarily performed to ensure proper stent expansion. In most cases, we used one stent. A longer stent was used when needed to ensure that the lesion was fully covered. Completion angiography was performed to determine the degree of residual stenosis. A technical success was defined as the absence of >30% residual stenosis.

The interventions were performed using dual antiplatelet agents (75 mg clopidogrel and 75 mg aspirin). The use of these agents was continued for six weeks after the operation and followed with aspirin monotherapy. A bolus of heparin (50 IU/kg) was administered during the procedure.

A neurological evaluation was independently performed after CAS by an experienced neurologist in all of the patients to determine whether minor or major strokes had occurred.

### Follow-up

A clinical examination and Doppler ultrasound (DUS) were performed immediately after CAS and at three, six, and 12 months and annually thereafter.

During these routine postoperative visits, the surgeon examined each patient, and a carotid duplex scan was obtained.

Moderate in-stent restenosis (>50% and <70%) was defined using DUS >250 cm/s as the peak systolic velocity (PSV) and a PSV ratio of the internal carotid artery (ICA) and the common carotid artery (CCA) that was less than four. Severe in-stent restenosis (>70%) was defined as a PSV >350 cm/s, an end-diastolic velocity >100 cm/s, and a PSV ratio of the ICA and the CCA of >4 [21].

### Endpoints

The primary endpoints were technical success, the presence of in-stent restenosis >50% or occlusion, either symptomatic or asymptomatic, during the follow-up period and risk factors for restenosis. The diagnosis and quantification of restenosis was noninvasively performed using DUS. DUS might over estimate and the severity of the lesion was therefore systematically confirmed using carotid CT angiography ([NASCET] criteria).

The secondary endpoints were early and late morbidity and mortality (TIA, stroke, myocardial infarction, or death).

A TIA was defined as the occurrence of any neurological deficit that completely resolved within 24 h

Any new neurological deficit that lasted for > 24 hours during the first 30 days was classified as a stroke. Major stroke was defined as a persistent and disabling neurological deficit that was present at the time of discharge. Minor stroke was defined as a persistent but nondisabling neurological deficit that was present at the time of discharge.

### Statistical methods

For categorical variables, the relationship between a variable and a primary endpoint was studied using chi-square tests or Fisher’s exact tests, when appropriate. The Mann-Whitney U test was used for continuous variables. A logistic regression model was used to estimate the odds ratio (OR) and 95% confidence interval (CI) of each predictor of restenosis. Only variables with a P-value <0.10 were considered eligible for the multivariate analysis, which was performed using a backward approach to control for possible confounding bias. Furthermore, to account for the potential of bias cause by loss to follow-up, the multivariate analysis was systematically adjusted for the duration of follow-up. The probability of restenosis was determine according to time, including the 95% CI, and obtained using the Kaplan-Meier method. All statistical tests were two-sided, and P-values <0.05 were considered to indicate statistical significance. All analyses were performed using R software (version 2.14.0).

## Results

### Demographic data

A total of 597 CAS procedures were performed at our institution between 1997 and 2013. Of these, 153 procedures were performed for post-CEA restenosis that were primarily caused by asymptomatic lesions (137/153). A total of 16 patients (10%) were treated for symptomatic lesions. During the same period, we performed 2063 CEA procedures and 47 redo surgeries for post-CEA restenosis.

Among the 153 cases, the initial surgery was eversion in 38 cases and standard carotid endarterectomy with a patch in 115 cases.

The average degree of restenosis was 82% (range, 80% to 95%) in asymptomatic patients and 81% (range, 60% to 99%) in symptomatic patients.

The characteristics of the patients are shown in Table 1.

**Table 1:** Baseline Characteristics.

	n = 153
**Age**	
Average, years	69.4
Minimum-maximum, years	36–89
Age > 70 years, n (%)	81 (53)
**Gender**	
Male, n (%)	114 (74)
**Cardiovascular risk factors, n (%)**	
Diabetes	41 (27)
Dyslipidemia	116 (76)
Hypertension	127 (83)
Active tobacco use	31 (20)
Previous tobacco use	88 (57)
Chronic kidney disease	24 (16)
**Antecedents to cardiovascular disease, n (%)**	
Coronary heart disease	79 (52)
Heart failure	10 (7)
Peripheral vascular disease	50 (33)
Stroke/transient ischemic attack	35 (23)
**Restenosis characteristics**	
Symptomatic, n (%)	16 (10)
Early < 24 months, n (%)	69 (45)
% stenosis (average, %)	82
**Contralateral lesions, n (%)**	
Occlusion	17 (11)
Stenosis > 50%	24 (16)

### CAS-related interventional parameters

Technical success was obtained in 98% of cases (150/153). The three technical failures consisted of two cases of residual stenosis over 30% and one failed catheterization of the CCA. The procedural data are shown in Table 2.

**Table 2:** Procedural data.

	n = 153
**Treatment, n (%)**	
Aspirin	151 (99)
Clopidogrel	153 (100)
Intraoperative heparin	153 (100)
**Technical characteristics, n (%)**	
Technical success	150 (98)
Cerebral protection system	125 (82)
FilterWire	124 (82)
Emboshield	1 (0.6)
Pre-dilation	13 (10)
Post dilation	144 (94)
**Number of stents, n (%)**	
1 stent	140 (91)
2 stents	11 (7.1)
3 stents	1 (0.5)
**Kind of stent, n (%)**	
Carotid Wall Stent	144 (94)
Cordis Precise Pro	5 (3)
Other	4 (3)

Of the 27 procedures (18%) in which no cerebral protection was provided, 25 were performed before 2003.

No procedures involved carotid angioplasty alone. In 92% (138/150) of the cases, we used only one stent. The average length of coverage was 38 mm. In most of the cases (94%-144/153), a closed-cell design Carotid WALLSTENT (Boston Scientific, Natick, Massachusetts) was used.

The external carotid artery remained patent in 95% of the cases (146/153).

### Perioperative morbidity and mortality

The 30-day perioperative stroke and death rate was 2.6% and included two TIAs and two minor strokes. The 30-day perioperative stroke and death rates for asymptomatic and symptomatic patients were 2.2% (3/137) and 6.2% (1/16), respectively. In the procedures that were performed without cerebral protection, the rate of neurological events was 7.4% (2/27).

The rate of major complications at 30 days was 3.3% and included four neurological events and one myocardial infarction. No deaths occurred during the initial 30-day follow-up period.

Ten minor perioperative complications (6.5%) were encountered, including 6 ICA spasms, 1 groin hematoma, 2 cases of mental confusion, and 1 case of bradycardia.

### Follow-up results

There were three technical failures, and they were excluded.

The average follow-up time was 36 months (range, 6–171 months) and the median follow-up was 26 months. In all, 100% of the patients (150/150) had DUS at the end of the follow-up period.

In-stent restenosis or thrombosis was observed in 16 patients (10.6%, 16/150), mainly during the first 24 months of follow-up (69%, 11/16). Twelve patients presented with restenosis, and four presented with occlusion. The average degree of restenosis was 81% (range, 50–95%). Symptomatic restenosis was observed in only one patient. Among the two cases of residual stenosis, neither involved in-stent restenosis.

**Fig 3:**
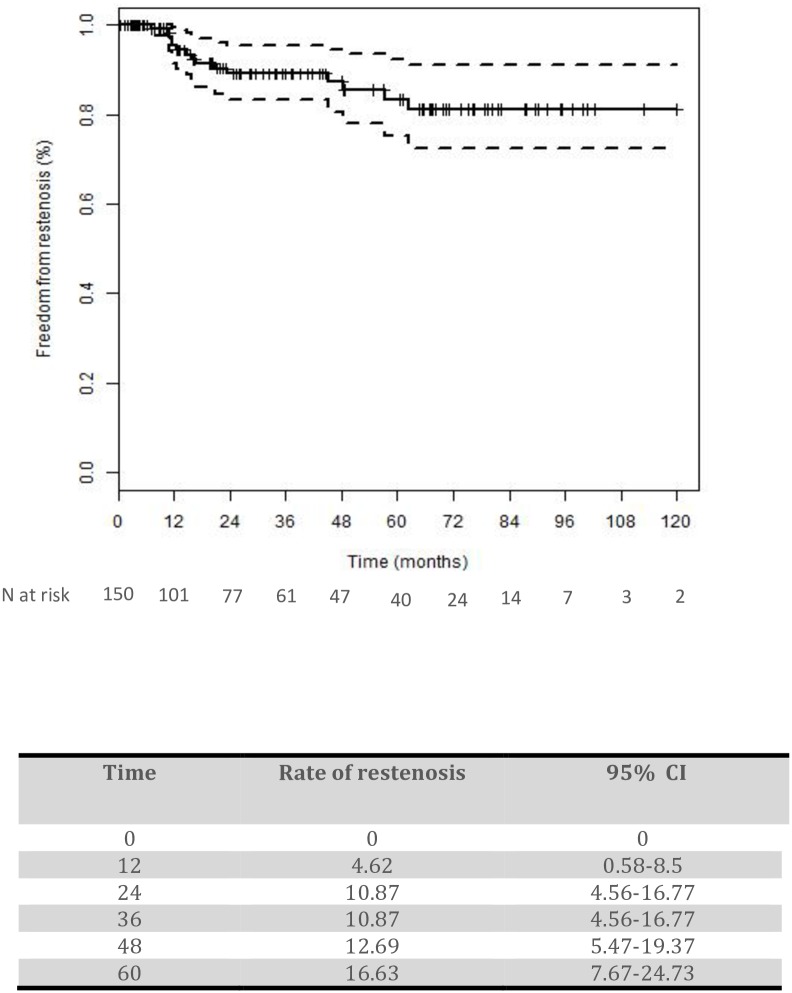
probability of in-stent restenosis or occlusion. Kaplan-Meier curve.

The probability of in-stent restenosis or occlusion was 4.62% (95% CI 0.58–8.5) at 12 months and 10.87% (95% CI 4.56–16.77) at 24 months (Fig 3).

**Fig 4:**
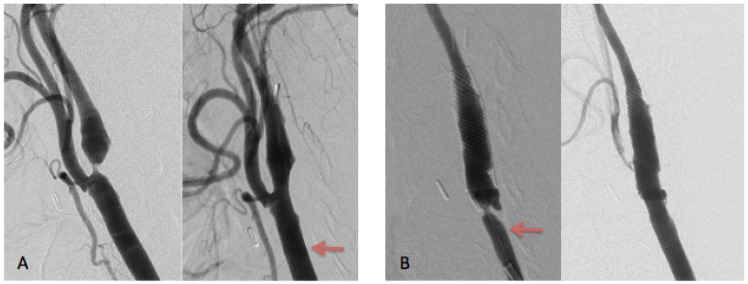
in-stent restenosis: restenosis on CCA after shortening of the carotid wall stent. A: CAROTID WALLSTENT 7–40 for a restenosis post CEA. B: shortening of the stent and early in-stent restenosis on CCA. The arrow is on the initial stent positioning.

Among the 12 cases of restenosis, eight occurred in the CCA, three in ICA1, and one in ICA3. The majority of restenoses (9/12) were located in the proximal area of the stent. In 3 cases, we observed a shortening of the Carotid WALLSTENT (Fig 4).

Eleven patients underwent carotid revascularization, including nine cases of iterative angioplasties (three that were angioplasties alone and five that were angioplasties with stenting) and two iterative surgeries (a vein graft). In one case (50% restenosis), a follow-up was performed using ultrasound.

The long-term mortality rate was 15.6% (23 patients). The cause of death was identified as cardiovascular in 39% of the cases (9/23). Specifically, there were four cases of myocardial infarction, one case of a ruptured abdominal aortic aneurysm, one case of mesenteric ischemia, one case of heart failure, and two cases of cardiovascular arrest. Seven patients died of lung cancer, four died of chronic obstructive pulmonary disease, and three died of unknown causes.

### Risk factors for restenosis

#### Univariate analysis

Younger age (< 70 years) and smoking were significantly associated with the onset of restenosis. There was no difference in the rate of CAS restenosis between early post-CEA restenosis and late post-CEA restenosis (Table 3).

**Table 3:** Demographic and Clinical Risk Factors: Univariate Analysis.

	Restenosis n = 16	No restenosis n = 134	P value
Gender (f), n (%)	3 (18.7)	35 (26.1)	0.76
Age, average (SD)	61.06 (8.8)	70.67 (9.24)	0.0003*
Age < 70 years, n (%)	14 (87.5)	56 (41.8)	0.0005*
Tobacco use (P/Y), average (SD)	39.3 (20)	28.8 (27)	0.041*
Coronary heart disease, n (%)	8 (50)	70 (52.2)	0.86
Diabetes, n (%)	2 (12.5)	38 (28.4)	0.23
HTN, n (%)	12 (75)	114 (85)	0.28
Hypercholesterolemia, n (%)	11 (68.7)	103 (76.8)	0.53
Statin, n (%)	10 (62.5)	96 (76.1)	0.23
Lesion characteristics			
Symptomatic	1 (6.2)	15 (11.1)	1
Early	6 (37.5)	60 (44.8)	0.57

*Statically significant

HTN = hypertension, py = pack years, SD = standard deviation

The analysis of technical factors revealed that there was a significant link between the extent of the area covered by the stent and the occurrence of anatomical events. A lack of stent coverage in the CCA was found to be a risk factor for restenosis (P = 0.027). We avoided covering the CCA in 17 cases (11.3%) in which the restenosis was at the distal end of the ICA (ICA2 and ICA3). Of the patients who did not have CCA coverage, 29.4% (5/17) developed restenosis, and 8.2% (11/133) of the patients who did have CCA coverage developed restenosis.

A high location (ICA3) was also significantly associated with the onset of restenosis (P = 0.020) (Table 4).

**Table 4:** Anatomical and Technical Factors: Univariate Analysis.

	Restenosis n = 16	No restenosis n = 124	P value
Stenosis > 85%, n (%)	10 (62.5)	51 (38.3)	0.06
Size ICA, average (SD)	5.36 (1.04)	5.54 (0.96)	0.98
Size CCA < 7.5 mm, n (%)	9 (56.2)	40 (32.2)	0.058
Calcification	0 (0)	6 (48.3)	1
Hypodensity	16 (100)	122 (98)	1
Ulceration	4 (25)	33 (26.6)	1
Dissection	0 (0)	3 (24.2)	1
Stenosis length, average (SD)	9.06 (5.4)	10.24 (5.25)	0.25
Location, n (%)			
CCA1	3 (18.7)	19 (15.3)	0.71
CCA2	3 (18.7)	17 (13.7)	0.70
ICA1	7 (43.7)	61 (49.1)	0.68
ICA2	3 (18.7)	43 (34.6)	0.20
ICA3	3 (18.7)	3 (2.4)	0.020*
Stent diameter, average (SD)	7.12 (0.89)	7.15 (0.81)	0.92
Stent length, average (SD)	36.88 (6.02)	37.04 (7.18)	0.82
ICA area covered, n (%)	15 (93.7)	114 (91.9)	1
CCA area covered, n (%)	11 (68.7)	112 (90.3)	0.027*
1 area covered, n (%)	6 (37.5)	22 (17.7)	0.09

*Statically significant

CCA = common carotid artery, ICA = internal carotid artery, SD = standard deviation

We also observed trends associated with anatomical characteristics. For example, a CCA diameter < 7.5 mm may increase the risk of restenosis (P = 0.058).

#### Multivariate analysis

Initial post-CEA restenoses were more severe in the group of patients who presented with an in-stent restenosis (OR: 5.11, 95% CI 1.31–19.92, P = 0.018). A lack of stent coverage of the CCA (OR: 7.9, 95% CI 1.78–35.05, P = 0.006) and younger age (< 70 years) (OR: 14.26, 95% CI 2.57–79.27, P = 0.002) were independent risks factors for in-stent restenosis (Table 5).

**Table 5:** Independent predictors of in-stent restenosis.

Variable	OR [CI 95%]	P Value
NASCET > 85	5.11 [1.31–19.92]	0.018*
Age < 70 Yrs	14.26 [2.57–79.27]	0.002*
CCA area not covered	7.9 [1.78–35.05]	0.006*
Time of follow-up	0.99 [0.97–1.01]	0.28

*Statically significant

OR = odds ratio, CI = confidence interval

## Discussion

In our study, treating restenosis after CEA with CAS was found to be a safe technique that yielded positive immediate, mid-term, and long-term results.

The ability to maintain positive anatomical results after CAS over the long term remains a concern. Patients treated for restenosis after CEA are more at risk for in-stent restenosis than patients treated for a primary lesion in a native artery [15, 16, 22–25].

This study spanned a long time period (1997–2013), and the CAS technique was uniformly applied throughout the study period, except that cerebral protection devices were introduced in 2003.

In our study, after an average follow-up period of 36 months, the incidence of in-stent restenosis was 10.6%. Based on the results of a Kaplan-Meier analyses, the incidence of in-stent restenosis was 10.87% (95% CI 4.56–16.77) after two years. These results show that in-stent restenosis may not limit the benefits of CAS for recurrent lesions as much as was previously thought; The rate of in-stent restenosis is similar to the rate of restenosis after redo surgery [8, 9, 26, 27]

The identification of risk factors for restenosis should improve the rate at which in-stent restenosis occurs. The risk factors for in-stent restenosis have previously been studied in heterogeneous populations [2, 17–19, 22, 23]. The originality of this study lies in our extensive examination of the anatomical (lesion evaluation) and technical risk factors (evaluation of the procedure) that are associated with restenosis in a homogenous population.

We identified new anatomical and technical risk factors for in-stent restenosis.

First, we found that as the severity (>85%) of the initial lesions increased, the risk of developing in-stent restenosis also increased. It is widely accepted that intimal and medial injuries that result from stent implantation induce a perivascular inflammatory response, and the severity of the induced arterial damage is correlated with the increase in inflammation [28], which leads to the stimulation of vascular smooth muscle cell proliferation and the development of in-stent restenosis [29]. We propose that a higher degree of trauma is induced by stent placement for severe lesions and that this trauma increases the inflammatory response[30, 31].

Second, we observed that a lack of stent coverage in the CCA was significantly associated with the risk of developing in-stent restenosis. Studies of coronary arteries have shown that there is an increased risk of in-stent restenosis at the site of stent inflow and, in the case of arterial bifurcation stenting [32, 33]. One explanation for this mode of development of in-stent restenosis is that arterial flow is disturbed and that the subsequent decrease in wall shear stress in the arterial bifurcation and at the site of stent inflow lead to the activation of growth factors, pro-inflammatory genes, and myointimal proliferation [32, 34].

Furthermore, in our study, the Carotid WALLSTENT (Boston Scientific, USA) was predominantly used, and we found that this self-expanding stent tends to shorten when placed in a bifurcation. Indeed, when the carotid bifurcation has a large diameter, the stent continuously tries to expand. In the case of a wide common carotid artery, it would perhaps be better to use a long, tapered, and self-expanding stent, such as the Acculink, X-act, or protege eV3, which are capable of satisfactorily adapting to the bifurcation.

Because carotid bifurcation is a high-risk area for restenosis, placing the proximal part of the stent in this area must be avoided.

Finally, anatomical risk factors may affect the long-term results of CAS. For example, a smaller CCA diameter (<7.5 mm) seems to be associated with an increased incidence of restenosis. This trend has already been reported for coronary arteries [35, 36].

Among the clinical variables that were analyzed in this study, only younger age (< 70 years) was found to be statistically significant in the multivariate analysis. Mousa et al found that age < 65 years old was a significant predictor of restenosis [37]. With regard for peripheral arterial disease, young patients, who likely have a more aggressive form of systemic atherosclerotic disease, appear to be predisposed to requiring multiple procedures or reinterventions [38]. We did find a significant link between smoking and in-stent restenosis in the univariate analysis, but these associates have been described in previous studies [39].

Based on our results, as a first line of treatment, we recommend covering the carotid bifurcation in cases of lesions in the internal proximal carotid artery (ICA 1 and ICA 2) to prevent in-stent restenosis. For lesions in ICA 3, we also recommend covering the bifurcation because we found an association in the univariate analysis that indicated that there is an increased risk of in-stent restenosis in lesions located on the distal carotid artery (ICA 3). The lack of coverage of the common carotid artery is one explanation for this association because 2 out of 3 patients who were treated for ICA 3 lesions were treated with only a stent in the ICA, and the restenosis in these patients was located in the bifurcation.

In the literature, anatomical and technical risk factors for restenosis include the number of stents, the presence of large and calcified lesions, and the existence of residual stenosis after the procedure. All of these risk factors were identified in heterogeneous populations or on primary lesions [17, 19]. In our study, these factors were not correlated with an increased risk of restenosis. These differences are most likely due to differences in the characteristics of the population being studied. The anatomical characteristics of recurrent lesions are very different from the characteristics of primary lesions because recurrent lesions are more often uniform with little calcification, and the bifurcation is therefore altered as a result of the initial surgery. In our population, only four patients had calcified lesions.

Our study was a retrospective analysis of prospectively gathered data that was analyzed in the context of a nonrandomized design project. The small number of observations of in-stent restenosis represents a potential weakness that limits the usefulness of the information provided in this study.

## Conclusion

We identified novel risk factors for in-stent restenosis that are specific to this population, and we propose a technical approach for reducing this risk.

We believe that a stent should cover the carotid bifurcation, especially when the Carotid WALLSTENT is used.

## Supporting Information

S1 File CAS for post CEA restenosis-xls. Statistical data.
(XLS)
